# Severe cutaneous adverse reactions associated with second-generation androgen receptor antagonists in prostate cancer patients

**DOI:** 10.1371/journal.pone.0325448

**Published:** 2025-06-10

**Authors:** Junfa Liu, Xiongfei Liu, Hongbo Zeng, Yangyang Tong, Zhe Chen, Zhitao Dong

**Affiliations:** 1 Department of Urology, The Second Xiangya Hospital, Central South University, Changsha, Hunan, China; 2 Department of Urology, Shenzhen Bao’an Traditional Chinese Medicine Hospital Group, Shenzhen, Guangdong, China; 3 Institute of Reproductive and Stem Cell Engineering, School of Basic Medical Science, Central South University, Changsha, China; 4 Department of Thoracic Surgery, The Second Xiangya Hospital, Central South University, Changsha, Hunan, China; West China Hospital of Sichuan University, CHINA

## Abstract

Prostate cancer ranks as the second most prevalent cancer among men, with androgen deprivation therapy (ADT) being a cornerstone treatment strategy. Enzalutamide, apalutamide, and darolutamide are key examples of second-generation androgen receptor antagonists (SGARAs). Although severe cutaneous adverse reactions (SCARs) are infrequent, they carry a significant risk of mortality. This study employed four disproportionality analysis algorithms: Reporting Odds Ratio (ROR), Proportional Reporting Ratio (PRR), Bayesian Confidence Propagation Neural Network (BCPNN), and Multi-item Gamma Poisson Shrinker (MGPS) to investigate the potential link between SGARAs and SCARs. As of the second quarter of 2024, reports of SCARs related to enzalutamide, apalutamide, and darolutamide totaled 25, 77, and 1, respectively. The majority of reports came from elderly patients, predominantly reported by health professionals, with Japan and the USA being the primary reporting countries. SCARs related to apalutamide detected positive signals in all four algorithms, while enzalutamide and darolutamide did not show positive signals. The study indicated that the majority of onset times occurred within 37 days, but SCARs could still occur up to 176 days with enzalutamide and 126 days after apalutamide treatment. No onset time was reported for darolutamide. In the treatment of prostate cancer with SGARAs, there is a potential risk of SCARs. When different SGARAs were compared, SCARs were more frequently reported with apalutamide than enzalutamide and darolutamide. This indicates that patients using SGARAs, particularly apalutamide, require closer and more prolonged monitoring to facilitate the early detection and management of SCARs and to reduce the occurrence of serious outcomes.

## Introduction

Prostate cancer, with approximately 1,500,000 new cases and 397,000 deaths globally in 2022, is the second most common cancer and the fifth most common cause of cancer deaths in men [1]. It is estimated that there will be approximately 2,900,000 new cases of prostate cancer worldwide in 2040, and approximately 700,000 deaths [2]. The etiology of prostate cancer remains incompletely understood, but established risk factors include advancing age, race and ethnicity, genetic mutations (such as BRCA1 and BRCA2), Lynch syndrome, and a family history of prostate cancer [3–7]. Additionally, metabolic syndrome, obesity, and smoking are also considered potential risk factors for prostate cancer [8]. Treatment options for prostate cancer include surgery, radiation therapy, chemotherapy, immunotherapy, androgen deprivation therapy (ADT), or a combination of these approaches

Androgen deprivation therapy is the cornerstone of prostate cancer treatment [9–11]. The fundamental approaches of ADT include inhibiting androgen production and blocking androgen-receptor binding. This can be done either by surgical or pharmacological castration. Surgical castration is the surgical removal of one or both testes to suppress androgen production. Pharmacological castration is related to androgens. The sex hormones exert their effects by binding to their receptors. Androgen receptor signaling inhibitors (ARSIs) are a group of drugs that block the androgen receptor pathway through various mechanisms: androgen synthesis inhibitors that block Cytochrome P450 17A1 (CYP17A1), such as abiraterone [12,13], and androgen receptor antagonists (ARAs), which include both steroidal (such as megestrol acetate, cyproterone acetate, and medroxyprogesterone) and nonsteroidal drugs (first-generation and second-generation androgen receptor antagonists) [11,14,15].

SGARAs include enzalutamide, apalutamide, and darolutamide, which were approved by the FDA for the treatment of prostate cancer in August 2012, February 2018, and July 2019, respectively. The three drugs differ in their chemical structures. Enzalutamide contains a 2-cyanophenyl moiety at the 3-position and a dimethyl moiety at the 5-position of the thiohydantoin ring. Apalutamide features a 2-cyanopyridine moiety at the 3-position and a spirocyclobutyl moiety at the 5-position of the thiohydantoin ring. Darolutamide (ODM-201) is a synthetic compound comprising a mixture (1:1) of two pharmacologically active diastereomers (ORM-16497 and ORM-16555). ODM-201 and its pharmacologically active main metabolite ORM-15341 are novel and structurally distinct from any known antiandrogens. Skin and subcutaneous tissue disorders are important adverse events of the three drugs [16–20]. Most cutaneous adverse reactions (CARs) caused by these drugs are mild to moderate with generally favorable outcomes, but a small number of patients may experience severe cutaneous adverse reactions (SCARs) [21–25]. SCARs are T-cell mediated, delayed drug hypersensitivity reactions (DHR), which are rare but can lead to multi-organ dysfunction and may be life-threatening. SCARs include drug reaction with eosinophilia and systemic symptoms(DRESS) or drug-induced hypersensitivity syndrome (DIHS), acute generalized exanthematous pustulosis (AGEP), and the most severe form, Stevens-Johnson syndrome and toxic epidermal necrolysis (SJS/TEN). Early identification of SCARs and discontinuation of the causative agent is extremely important to avoid disease progression [26–28].

Disproportionality analysis is a quantitative method by comparing the frequency of adverse event reports for a specific drug with the frequency of reports for the same adverse event associated with other drugs, to detect differences that may indicate associations between the drug and the adverse reactions [29]. The four commonly used algorithms in disproportionality analysis are Reporting Odds Ratio (ROR), Proportional Reporting Ratio (PRR), Bayesian Confidence Propagation Neural Network (BCPNN), and Multi-item Gamma Poisson Shrinker (MGPS). ROR and PRR are frequentist (non-Bayesian) algorithms, while BCPNN and MGPS are Bayesian algorithms [30–33]. The advantages of non-Bayesian algorithms are their simplicity in calculation and high sensitivity. However, when the number of adverse events is low, these algorithms are prone to false-positive adverse events signals. The advantages of Bayesian algorithms are their stability and ability to reduce false-positive adverse events signals. However, they are computationally complex and have a relatively delayed signal detection time [34]. In this study, four algorithms were used together to minimize the errors caused by using a single algorithm.

## Methods

**Fig 1:**
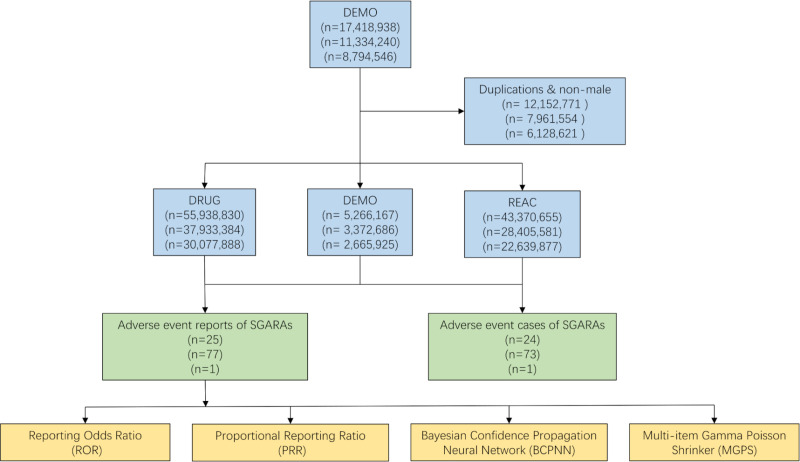
The flow diagram of selecting SGARAs-related AEs from FAERS database.

The Food and Drug Administration Adverse Event Reporting System (FAERS) database is a publicly available global database with data from spontaneous reports from health professionals and non-health professionals. The FAERS database is composed of the following seven components: demographics, indications, drugs, therapies, reactions, outcomes, and report sources. In this study, data were collected on enzalutamide, apalutamide, and darolutamide as primary suspects (PS), SCARs as preferred term (PT), limited to men with prostate cancer. The data covers the period from the FDA approval date of each of the three drugs up to the second quarter of 2024. Data collection and cleaning were performed using RStudio. The FAERS database collected 17,418,938, 11,334,240, 8,794,546 reports, and 12,152,771, 7,961,554, 6,128,621 reports were removed according to FDA guidelines. When CASEID is the same, the latest FDADT was selected. If both CASEID and FDADT are identical, the higher PRIMARYID was retained [35]. Fig 1 illustrates the process for selecting SGARAs-related AEs based on the FAERS database. Disproportionality analysis was performed using ROR, PRR, BCPNN, MGPS. The parameters required for these four algorithms are calculated based on a 2 × 2 table, as shown in Table 1. The formulas for each method and conditions for signal generation are detailed in Table 2.

**Table 1:** Four grid table.

	Target ADEs	Non-target ADEs	Total
SGARA	a	b	a + b
Non-SGARA	c	d	c + d
Total	a + c	b + d	N = a + b + c + d

a, number of reports containing both the target drug and target adverse drug reaction; b, number of reports containing other adverse drug reaction of the target drug; c, number of reports containing the target adverse drug reaction of other drugs; d, number of reports containing other drugs and other adverse drug reactions; N, number of reports.

**Table 2:** Four major algorithms used for signal detection.

Algorithms	Equation	Criteria
ROR	ROR = ad/b/c	lower limit of 95% CI > 1, a ≥ 3
	95%CI = e^ln(ROR)±1.96(1/a + 1/b + 1/c + 1/d)^0.5^	
PRR	PRR = a(c + d)/c/(a + b)	PRR ≥ 2, χ^2^ ≥ 4, a ≥ 3
	χ^2^ = exp(2 * log(abs(a * d – b * c)) + log(a + b + c + d) – log(a + b) + log(c + d) + log(a + c) + log(b + d))	
BCPNN	IC = log_2_a(a + b + c + d)(a + c)(a + b)	IC025 > 0
	95%CI = E(IC) ± 2V(IC)^0.5	
MGPS	EBGM = a(a + b + c + d)/(a + c)/(a + b)	EBGM05 > 2
	95%CI = e^ln(EBGM)±1.96(1/a + 1/b + 1/c + 1/d)^0.5^	

Abbreviations: ROR, reporting odds ratio; 95% CI, 95% confidence interval; PRR, Proportional Reporting Ratio; χ^2^, chi-squared; BCPNN, bayesian confidence propagation neural network; IC, information component; E(IC), the IC expectations; V(IC), the variance of IC; IC025, the lower limit of 95% CI of the IC; MGPS, Multi-item Gamma Poisson Shrinker; EBGM, empirical Bayesian geometric mean; EBGM05, the lower limit of 95% CI of EBGM.

## Results

### Descriptive analysis

Because a patient may simultaneously experience one or more adverse reactions belonging to the SCARs, we use the term “cases” to refer to the number of patients who experienced SCARs after using SGARAs, and “reports” to denote the number of adverse reactions caused by SGARAs. After data cleaning, 24 cases and 25 reports for enzalutamide, 73 cases and 77 reports for apalutamide, and 1 case and 1 report for darolutamide were included in the final analysis. Table 3 provides a detailed overview of the patient demographics and clinical features for SCARs cases reported in FAERS. Of the three drugs, the highest number of SCARs cases were reported for apalutamide, followed by enzalutamide, and the least for darolutamide. Elderly patients (≥65 years) comprised the majority of SCARs cases for enzalutamide and apalutamide, at 70.8% and 57.5%, respectively, with two SCARs cases in apalutamide patients under 18 years old. The only patient reporting SCARs for darolutamide was under 18 years old. Adverse reactions were primarily reported by health professionals, with 16 (66.7%), 64 (87.7%), and 1 (100%) cases for the three drugs, respectively. The highest number of cases came from Japan (41.7%, 38.4%, 100%), followed by the United States (25.0%, 15.1%, 0%), with these two countries contributing over half of the total cases.

**Table 3:** Patient characteristics of SCARs with SGARAs in the FAERS database.

Characteristics	Enzalutamide	Apalutamide	Darolutamide
Age group[n(%)]			
<18	0 (0%)	2 (2.7%)	1 (100%)
18–64	4 (16.7%)	5 (6.8%)	0 (0%)
65–85	17 (70.8%)	42 (57.5%)	0 (0%)
>85	0 (0%)	8 (11%)	0 (0%)
Missing	3 (12.5%)	16 (21.9%)	0 (0%)
Reporter			
Health Professionals	16 (66.7%)	64 (87.7%)	1 (100%)
Non-Health Professionals	8 (33.3%)	7 (9.6%)	0 (0%)
Missing	0 (0%)	2 (2.7%)	0 (0%)
Reported Countries			
JP	10 (41.7%)	28 (38.4%)	1 (100%)
US	6 (25.0%)	11 (15.1%)	0 (0%)
FR	2 (8.3%)	7 (9.6%)	0 (0%)
TW	2 (8.3%)	2 (2.7%)	0 (0%)
GB	1 (4.2%)	1 (1.4%)	0 (0%)
BR	1 (4.2%)	0 (0%)	0 (0%)
IT	1 (4.2%)	0 (0%)	0 (0%)
CN	0 (0%)	8 (11%)	0 (0%)
DE	0 (0%)	3 (4.1%)	0 (0%)
KR	0 (0%)	3 (4.1%)	0 (0%)
IE	0 (0%)	2 (2.7%)	0 (0%)
FI	0 (0%)	2 (2.7%)	0 (0%)
CH	0 (0%)	2 (2.7%)	0 (0%)
BE	0 (0%)	1 (1.4%)	0 (0%)
NL	0 (0%)	1 (1.4%)	0 (0%)
RO	0 (0%)	1 (1.4%)	0 (0%)
COUNTRY NOT SPECIFIED	1 (4.2%)	1 (1.4%)	0 (0%)

Abbreviations: JP, Japan; US, the United States of America; FR, the French Republic; TW, Taiwan (Province of China); GB, the United Kingdom of Great Britain and Northern Ireland; BR, the Federative Republic of Brazil; IT, the Republic of Italy; CN, the People’s Republic of China; DE, the Federal Republic of Germany; KR, the Republic of Korea; IE, Ireland; FI, the Republic of Finland; CH, the Swiss Confederation; BE, the Kingdom of Belgium; NL, the Kingdom of the Netherlands; RO, Romania.

### Disproportionality analysis

The number of SCARs reports linked to enzalutamide, apalutamide, and darolutamide was 25, 77, and 1, respectively. Table 4 presents the findings of the disproportionality analysis conducted using four distinct methods. Positive signals for SCARs were detected in all four algorithms for apalutamide, while no positive signals were detected for enzalutamide or darolutamide in any of the four algorithms.

**Table 4:** Signal strength of SCARs related to SGARAs.

Drugs	reports	ROR (95% CI)	PRR (χ^2^)	IC (IC025)	EBGM (EBGM05)
Enzalutamide	25	0.36 (0.23–0.55)	0.36 (24.7)	−1.14 (−1.51)	0.45 (0.3)
Apalutamide	77	33.32 (23.37–47.49)	33.04 (953.71)	3.78 (3.64)	13.76 (9.66)
Darolutamide	1	0.43 (0.06–3.07)	0.43 (0.75)	−1.2 (−3.19)	0.43 (0.06)

Abbreviations: ROR, reporting odds ratio; 95% CI, 95% confidence interval; PRR, Proportional Reporting Ratio; χ^2^, chi-squared; IC, information component; IC025, the lower limit of 95% CI of the IC; EBGM, empirical Bayesian geometric mean; EBGM05, the lower limit of 95% CI of EBGM.

### Time-to-Onset analysis

**Fig 2:**
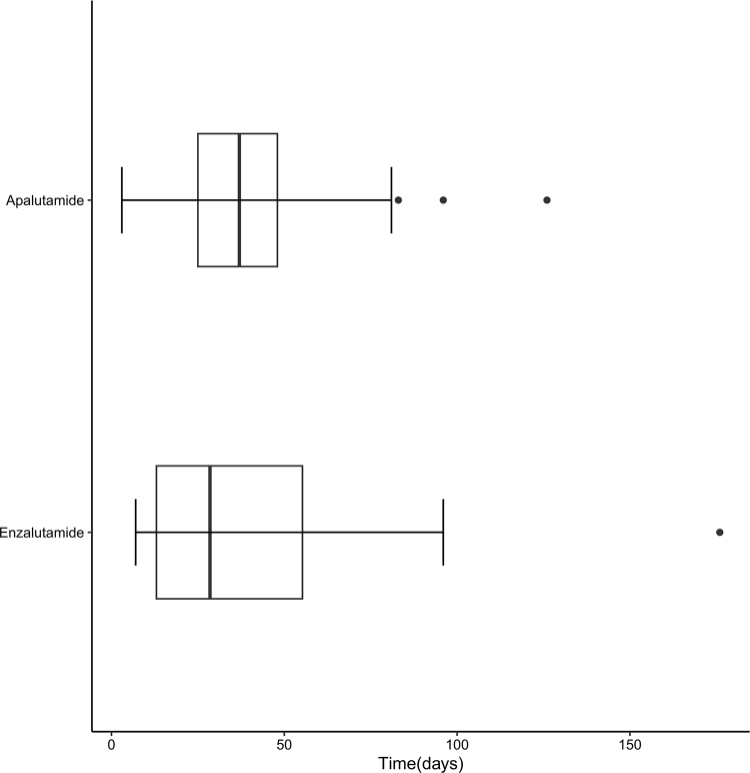
Onset times for SCARs associated with SGARAs.

There were 14 and 41 reported cases of SCARs associated with enzalutamide and apalutamide, respectively. The results are illustrated in Fig 2. The majority of onset times of SCARs with enzalutamide (64.3%) and apalutamide (51.2%) occurred within 37 days. However, SCARs can still occur after 176 days with enzalutamide (7.1%) and 126 days with apalutamide (2.4%). The median time to onset for enzalutamide and apalutamide was 28.5 days (interquartile range 11–60 days) and 30 days (interquartile range 22–48 days), respectively. No onset time data were reported for SCARs associated with darolutamide.

## Discussion

The amount of data remaining after our cleaning process is less than that reported by others, and we believe these differences are due to variations in the selection criteria applied during data selection [36–38]. The indications listed in the labels for the three drugs are prostate cancer. However, in the database, the indications for these drugs also include ‘product used for unknown indication’ and ‘malignant neoplasm’. To enhance the reliability of our data, we restricted the indications for the three drugs to prostate cancer during the cleaning process, excluding data with other indications. Additionally, the role of the drug was limited to being the primary suspect in our study. Therefore, 35,236, 3,562, and 995 patients treated with enzalutamide, apalutamide, and darolutamide, respectively, were included in our study.

The results indicate that among the patients with SCARs, three were under the age of 18. Prostate cancer is very rare in children and young adults. The three cases were reported by physicians from Japan. A Japanese scholar published a case report of an 11-year-old boy with prostate cancer [39]. Furthermore, we found multiple reports of prostate cancer in children and young adults, some of which involved patients undergoing anti-androgen therapy [40–49]. Therefore, although prostate cancer in individuals under 18 years old is rare, there are cases documented in the literature. The results indicate that the majority of reports come from elderly patients, which may be related to the age distribution of prostate cancer incidence. According to U. S. cancer statistics, the incidence of prostate cancer is age-related, with the incidence of prostate cancer in men increasing from 0.2% at birth to 49 years of age, all the way up to 9.0% at 70 years of age and older [50]. The FDA approved enzalutamide, apalutamide, and darolutamide for prostate cancer in 2012, 2018, and 2019, respectively. These drugs initially marketed in the United States and then gradually rolled out in other countries. Consequently, the majority of reports come from Japan and the United States, likely due to the earlier market introduction and higher awareness of drug adverse event reporting in these countries.

Previous clinical trials have shown that apalutamide has a higher incidence of skin and subcutaneous tissue disorders compared to enzalutamide and darolutamide [17,19,20]. The disproportionality analysis results show that positive signals were detected only for apalutamide across all four algorithms, with strong signal intensities. This indicates that the use of apalutamide in prostate cancer is associated with an increased risk of SCARs compared to other medications, which is consistent with the clinical trial results. Studies suggest that the 2-cyanopyridine moiety in apalutamide may react with cysteine in proteins forming haptens, which could trigger an immune response, leading to skin and subcutaneous tissue disorders. The 2-cyanopyridine in apalutamide is replaced by 2-cyanophenyl in enzalutamide, which may explain the lower incidence of skin and subcutaneous tissue disorders observed with enzalutamide compared to apalutamide [51]. Darolutamide features distinct structural attributes when compared to enzalutamide and apalutamide. It has two pharmacologically active diastereomers, lower blood-brain barrier permeability, and lacks CYP pathway-mediated effects, it is associated with the fewest grade 3 + adverse events among SGARAs for managing non-metastatic castration-resistant prostate cancer [52,53]. At the same time, darolutamide is the latest to market, which may explain the lower number of adverse event reports, including only one SCARs report.

Up to 2% of drug eruptions, referred to as SCARs, progress rapidly and can lead to multi-organ dysfunction or even death [54]. Health professionals, such as physicians, registered nurses, pharmacists, and dentists, who are directly involved in patient care and treatment, possess specialized medical knowledge and clinical experience. They are the primary individuals responsible for identifying SCARs. As shown in the results of this study, health professionals serve as the principal reporters. Meanwhile, since early recognition and treatment are crucial for improving outcomes in patients with SCARs, enhancing the ability of non-health professionals to identify SCARs is also important [55]. If SCARs are suspected or confirmed as a drug adverse reaction, discontinuation of the suspected drug and avoidance of re-administration are the primary measures. Treatment should include symptomatic and supportive care to maintain fluid and electrolyte balance, prevent infections, use glucocorticoids to suppress inflammation and immune-mediated tissue damage, intravenous immunoglobulin, oral cyclosporine to suppress immune function and apoptosis, and plasmapheresis [56–58]. Although disproportionality analysis for enzalutamide and darolutamide was negative, these drugs may still cause SCARs, and vigilance is required in clinical practice. Adverse reactions for enzalutamide and apalutamide generally occur within 37 days, but SCARs may still manifest after 126 days or longer, this underscores the need for more intensive monitoring of patients on SGARAs, especially during the first 37 days, with ongoing long-term follow-up also being crucial. Further research with extended follow-up and larger datasets is required to elucidate the relationship between SGARAs and SCARs.

Due to the inherent limitations of the FAERS database, disproportionality analysis cannot be used alone to evaluate and describe a drug’s safety profile. The spontaneous nature of reporting can result in under-reporting or over-reporting, leading to reporting bias and affecting overall data quality [59,60]. The data in the FAERS database are reported by both medical professionals and non-medical personnel. The absence of medical expertise among non-medical reporters and the lack of verification may lead to potential inaccuracies in the data. The FAERS database collects a part of cases with adverse events, as the total number of patients using the drug is unknown. Therefore, performing disproportionality analysis in the FAERS database does not allow for the calculation of incidence rates or reporting rates, nor can it infer causal relationships between the drug and adverse events or assess the relative risk of adverse events between different drugs [61–63]. Consequently, additional studies, such as prospective clinical trials and observational studies are necessary to obtain a more complete understanding of a drug’s safety profile.

## Conclusion

In conclusion, SGARAs are widely used in the treatment of prostate cancer with good efficacy, but their adverse effects cannot be ignored, especially DRESS/DIHS, AGEP, and SJS/TEN can result in serious outcomes, causing death in severe cases. Our findings indicate that SGARAs are associated with increased reporting of SCARs among prostate cancer patients. In clinical practice, there is a need to be alert to the occurrence of SCARs and to reduce the occurrence of serious outcomes. Meanwhile, further studies with large sample sizes are needed to validate the association between SGARAs and SCARs.
